# The Importance of Autoantibody Detection in Primary Biliary Cirrhosis

**DOI:** 10.3389/fimmu.2015.00309

**Published:** 2015-06-23

**Authors:** Eduardo Luiz Rachid Cancado, Michelle Harriz

**Affiliations:** ^1^Department of Gastroenterology, Clinical Gastroenterology and Clinical Hepatology of Hospital das Clinicas, University of São Paulo School of Medicine, São Paulo, Brazil; ^2^Laboratory of Immunopathology of Schistosomiasis, Institute of Tropical Medicine, University of São Paulo, São Paulo, Brazil; ^3^Laboratory of Tropical Gastroenterology and Hepatology, Institute of Tropical Medicine, São Paulo, Brazil

**Keywords:** primary biliary cirrhosis, antimitochondrial antibodies, antinuclear antibodies, autoimmune cholangitis, autoimmune liver diseases

The role of autoantibodies in primary biliary cirrhosis (PBC) is not only to aid in the diagnosis of this disease but also to classify and assist in defining its prognosis. For the diagnosis of PBC, the patient must have at least two of the following three parameters: clinical and/or biochemical characteristics of cholestasis, reactivity of anti-mitochondrial antibodies, and histological changes associated with cholestasis, particularly florid biliary lesions and portal granulomas.

There are two methods to detect AMAs. Indirect immunofluorescence (IIF) is the most common method, using unfixed sections of the kidney and stomach from rodents as a substrate. The presence of a fluorescent pattern in the cortical regions, and specifically in the medullary renal regions and in the proximal, distal, and collecting tubules, is very characteristic of these antibodies (Figure 1A). A concomitant staining in gastric parietal cells is usually observed. Alternatively, reactivity against purified or recombinant antigens derived from the multi-enzyme 2-oxoacid dehydrogenase complex (2-OADC) is observed, which consists of the pyruvate dehydrogenase complex, oxoglutarate dehydrogenase complex, and branched-chain oxoacid dehydrogenase complex (particularly against epitopes on their E2 subunits, which contain lipoic acid, a co-factor of these enzymes). With this antigenic source, anti-2OADC antibodies can be detected using immunoblotting, immunodiffusion, ELISA, and the Line immunoassay. Anti-M2 nomenclature for these antibodies should be avoided because it uses an old classification of AMAs that has not yet been proven. A characteristic cytoplasmic staining pattern observed by IIF when testing sera for antinuclear antibodies (ANAs) called the “strand of beads” suggests the presence of AMAs (Figures 1B,C). Technical professionals who perform the detection of autoantibodies and medical doctors who receive the results should adequately examine the patient. The presence of this pattern needs to be confirmed using a specific technique because it does not necessarily indicate AMA reactivity.

**Figure 1:**
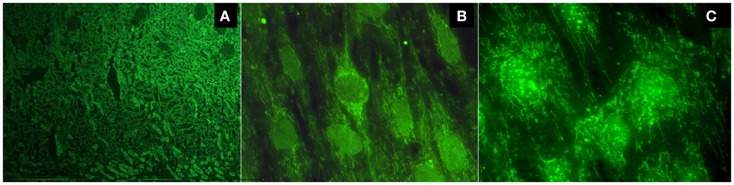
**(A)** Typical fluorescent pattern of anti-mitochondrial antibodies reacting against the proximal, distal, and collecting tubules, leaving the glomeruli unstained; **(B)** the cytoplasmic pattern "strand of beads" and the nuclear envelope pattern; **(C)** multiple nuclear dot pattern.

AMAs can be detected in over 90% of patients with PBC. In the vast majority of patients (90–95%), this reactivity is against the E2 subunit of the pyruvate dehydrogenase complex (74 kDa band by immunoblotting). In addition, 50–80% of PBC patients react against the E2 subunit of the branched chain oxoacid dehydrogenase complex (52 kDa) and 20–60% react against the E2 subunit of the oxoglutarate dehydrogenase complex (48 kDa). Less frequently, the reactivity is against the E1a (40 kDa) and E3 binding protein subunits (50 kDa) of the pyruvate dehydrogenase complex (1, 2). Testing serum samples by ELISA or immunoblotting is important to confirm dubious fluorescent patterns or negative sera by IIF. In this context, by investigating the AMAs by IIF and complementing with ELISA and immunoblotting, the diagnosis can be confirmed in 95% of patients with clinical and histological features of PBC. In our experience, immunoblotting is the best technique to study anti-2OADC antibodies.

Although AMA reactivity is highly suggestive of PBC, the specificity of its presence for this diagnosis is approximately 90%. AMA reactivity can be observed in AIH (5–10% of all AMA reactive sera or 5% of patients with AIH) and characterizes a variant form of this disease without showing other features of an overlapping syndrome. Its reactivity can also be detected in some patients with chronic hepatitis C, as well as in individual family members who are screened from a diagnosed patient with PBC, and in patients tested for ANAs with rheumatological diseases and normal values of alkaline phosphatase. Under these conditions, a diagnosis of PBC cannot be defined without a confirmatory liver biopsy (it is necessary to observe the presence of at least two parameters as previously described). Even when a liver biopsy is performed, the histological features of PBC are not always present; thus, its diagnosis is not achieved. Nevertheless, some patients will show features of the disease.

There are controversies if the AMA titers are related to disease severity or disease progression. Dellavance et al. tested the sera from four different groups with AMA reactivity, such as samples obtained from patients with a definite diagnosis of PBC with or without associated autoimmune diseases and from individuals with AMA reactivity and normal biochemical tests with or without associated autoimmune diseases. Patients who exhibited triple isotype AMA reactivity (IgG/IgM/IgA) by IIF, higher levels of anti-pyruvate dehydrogenase complex by ELISA, higher anti-pyruvate dehydrogenase avidity, and multiple antibody panel reactivity had a more definite diagnosis of PBC with or without associated autoimmune diseases. According to this study, the autoantibody profile was quantitatively and qualitatively more robust in patients with a definite diagnosis of PBC (3). Evidence that the reactivity of AMA has some relationship with the activity of the liver disease is derived from the observation that patients in the early stage of PBC treated with ursodeoxycholic acid can become AMA-negative, but this event is uncommon (4). The strongest evidence that favors a causal relationship between the pathogenesis of PBC and AMA reactivity is derived from experimental models of PBC; for example, one of the female SJL mice demonstrates a breakdown in tolerance against PDC antigens with anti-PDC antibody production followed by PBC-like biliary duct lesions, which is also known as experimental autoimmune cholangitis (5). There are other interesting models of the experimental induction of AMA reactivity and the development of PBC-like biliary duct lesions following the administration of xenobiotics in guinea-pigs and mice (2, 6).

Antinuclear antibodies are frequently detected in PBC (in approximately 50% of patients). Two patterns of ANAs are highly specific: the nuclear envelope, the whose main target antigens of which are gp210, p62, and the lamin B receptor, and multiple nuclear dots, the main target antigens of which are sp100, sp140, and promyelocytic leukemia protein (PML). Each of these patterns can be observed in approximately 20–40% of patients (Figures 1B,C). Anti-centromere antibodies tested positive in 10–30% of PBC patients and are frequently detected in patients with a limited form of systemic scleroderma or Sjögren’s syndrome. In PBC patients without these diseases, their reactivity could represent pre-clinical markers of these diseases (1).

In the vast majority of patients, the reactivity of ANAs is present together with AMA reactivity. Nevertheless, close to 5% of patients with clinical, biochemical, or histological features of PBC have ANA reactivity detected alone and are classified as AMA-negative PBC or autoimmune cholangitis. It is questionable whether patients with AMA reactivity have different clinical manifestations from those without AMA reactivity. However, itching was less frequently observed in AMA-negative patients, and the levels of alkaline phosphatase and IgM were lower in patients with these serological features. By contrast, the bile duct damage around the portal area was milder in AMA-reactive patients (6, 7).

Even in patients with both reactivity to AMA and ANA, there is some evidence that those with ANA reactivity have a different clinical behavior. As previously reported in several studies, the persistence of reactivity of anti-gp210 is a strong risk factor for the evolution to end-stage liver disease. Patients with anti-gp210 reactivity had more severe interface hepatitis, lobular inflammation, and ductular reactions. Moreover, the prognosis was more favorable for patients who were initially positive for anti-gp210 and became negative during the course of therapy (1, 8–11).

Evidence suggesting that patients with reactivity for anti-sp100 antibodies who have a more severe progressive disease is less convincing than the evidence for anti-gp210. Interestingly, the reactivity for these antibodies was more commonly described in PBC patients who experienced a urinary tract infection (74 versus 4.8%) (12). In relationship to anti-centromere antibodies, the results regarding this issue are inconclusive. However, some studies have suggested a relationship between anti-centromere reactivity and the development of cirrhosis and portal hypertension (1, 13).

In a study with Brazilian patients, AMAs and/or anti-2OADC antibodies are disease markers close to 96%, and 4% of patients have isolated reactivity against the nuclear envelope and/or nuclear body proteins (the so-called AMA-negative PBC or autoimmune cholangitis). Anti-centromere antibodies were detected in 17.7% of all patients and in 32.4% of patients with extra-hepatic autoimmune diseases, such as systemic scleroderma and/or Sjögren syndrome. Among the 130 patients who tested positive for specific antibodies against nuclear antigens, 22.3 and 25.4% of patients demonstrated reactivity for nuclear envelope proteins and multiple nuclear dots, respectively (3). These results are very similar to those obtained at other centers (1, 2).

The overlap of PBC-specific antibodies is currently easier to record due to the variety of commercially available ELISA assays. In Liu’s study in 2010, 922 PBC patients with AMA reactivity by IIF were tested using IgG/IgA dual isotype ELISA for detecting multiple mitochondrial antibodies (against recombinant antigens of the three enzymes of 2-OADC – MIT3) and nuclear autoantibodies specific for PBC (anti-sp100 and anti-gp210) (14). In patients with AMA-positive PBC, 92.4% were also positive for anti-MIT3 and approximately 20% were positive for anti-gp210 and anti-sp100. Eight-hundred-sixty-eight (94.1%) patients were positive using one or more specific ELISAs. However, the sera of 16 (1.8%) patients demonstrated reactivity for only sp100 or gp210 antigens. By contrast, in 253 patients with AMA-negative PBC, 28.1 and 15% of patients were positive for anti-MIT3 and 15 were positive for anti-gp210 or anti-sp100, respectively. Furthermore, 117 (46.2%) patients were positive by one or more specific ELISAs. However, 46 patients (39.3%) demonstrated reactivity to only sp100 and/or gp210. The conclusion of this study was that the detection of anti-MIT3 was valuable in AMA-negative PBC by IIF.

Another method used to detect the overlap of PBC-related antibodies is the line immunoassay. Using this technique, several antigens are immobilized on strips and are incubated with serum samples obtained from patients with suspected autoimmune liver disease (15). In general, these commercial assays for the simultaneous detection of several antibodies are expensive, and the IIF technique continues to be the best method to initiate the investigation of liver diseases because it simultaneously tests several autoimmune liver disease-related autoantibodies.

In summary, the detection of autoantibodies for the diagnosis of PBC should follow this algorithm. Patients with chronic cholestasis should be tested for AMA using IIF. Patients with reactivity are termed AMA-positive PBC patients, and patients without chronic cholestasis should be tested against 2-OADC antigens using immunoblotting or ELISA. Patients with any reactivity also have AMA/anti-2OADC antibody-positive PBC. Patients without reactivity to AMA and anti-2OADC antibodies should be tested for specific ANAs, particularly against nuclear bodies and nuclear envelope proteins by IIF or ELISA. Patients with any isolated reactivity have AMA-negative PBC or autoimmune cholangitis. Although PBC can be diagnosed without reactivity to AMA, anti-2-OADC, or nuclear antigens according to international criteria, we prefer to designate these patients as having non-PBC cholestasis. Although there are controversies, the prognosis of liver disease can be correlated to the ANA pattern.

## Conflict of Interest Statement

The authors declare that the research was conducted in the absence of any commercial or financial relationships that could be construed as a potential conflict of interest.
